# Novel Silver-Functionalized Poly(ε-Caprolactone)/Biphasic Calcium Phosphate Scaffolds Designed to Counteract Post-Surgical Infections in Orthopedic Applications

**DOI:** 10.3390/ijms221810176

**Published:** 2021-09-21

**Authors:** Sara Comini, Rosaria Sparti, Bartolomeo Coppola, Mehdi Mohammadi, Sara Scutera, Francesca Menotti, Giuliana Banche, Anna Maria Cuffini, Paola Palmero, Valeria Allizond

**Affiliations:** 1 Bacteriology and Mycology Laboratory, Department of Public Health and Pediatrics, University of Torino, Via Santena 9, 10126 Turin, Italy; sara.comini@unito.it (S.C.); francesca.menotti@unito.it (F.M.); annamaria.cuffini@unito.it (A.M.C.); valeria.allizond@unito.it (V.A.); 2 Immunology Laboratory, Department of Public Health and Pediatrics, University of Torino, Via Santena 9, 10126 Turin, Italy; rosaria.sparti@unito.it (R.S.); sara.scutera@unito.it (S.S.); 3 INSTM R.U. Lince Laboratory, Department of Applied Science and Technology, Politecnico di Torino, Corso Duca degli Abruzzi, 24, 10129 Turin, Italy; bartolomeo.coppola@polito.it (B.C.); mehdi.mohammadi@polito.it (M.M.); paola.palmero@polito.it (P.P.)

**Keywords:** prosthetic joint infections, poly(ε-caprolactone), scaffolds, biphasic calcium phosphate, silver ions, *Staphylococcus aureus*, antibacterial properties, Saos-2 cell vitality/proliferation

## Abstract

In this study, we designed and developed novel poly(ε-caprolactone) (PCL)-based biomaterials, for use as bone scaffolds, through modification with both biphasic calcium phosphate (BCP), to impart bioactive/bioresorbable properties, and with silver nitrate, to provide antibacterial protection against *Staphylococcus aureus*, a microorganism involved in prosthetic joint infections (PJIs). Field emission scanning electron microscopy (FESEM) showed that the samples were characterized by square-shaped macropores, and energy dispersive X-ray spectroscopy analysis confirmed the presence of PCL and BCP phases, while inductively coupled plasma–mass spectrometry (ICP–MS) established the release of Ag^+^ in the medium (~0.15–0.8 wt% of initial Ag content). Adhesion assays revealed a significant (*p* < 0.0001) reduction in both adherent and planktonic staphylococci on the Ag-functionalized biomaterials, and the presence of an inhibition halo confirmed Ag release from enriched samples. To assess the potential outcome in promoting bone integration, preliminary tests on sarcoma osteogenic-2 (Saos-2) cells indicated PCL and BCP/PCL biocompatibility, but a reduction in viability was observed for Ag-added biomaterials. Due to their combined biodegrading and antimicrobial properties, the silver-enriched BCP/PCL-based scaffolds showed good potential for engineering of bone tissue and for reducing PJIs as a microbial anti-adhesive tool used in the delivery of targeted antimicrobial molecules, even if the amount of silver needs to be tuned to improve osteointegration.

## 1. Introduction

Orthopedic prosthetic surgery for bone repair is the most common standard cure worldwide for joint osteoarthritis, rheumatic diseases, tumor resection and some types of fractures [1,2,3,4]. Currently, physicians perform millions of these surgeries, but the number is expected to quadruple over the next one to two decades [5,6,7]. One contributor to this trend is the increase in bone replacements due to populations becoming increasingly older and overweight [8,9,10]. 

Prosthetic joint infection (PJI) represents the second most frequent (~2%), but also the most important, complication of orthopedic prosthetic surgery after aseptic loosening [8,10,11,12,13]. The current rate of PJIs varies from one center to another. Sabah et al. reported that infection is an indication for surgery in 10–15% of first revision procedures, which is in line with Italian data; however, this prevalence is much greater in revisions, estimated to be around 23% overall [5,14,15,16]. The main concern of a diagnostic evaluation is the accurate differentiation between septic and aseptic failure because the treatment is different. Regarding PJIs, both diagnosis and treatment are challenging [10,11]. The diagnosis of PJIs still remain a challenge because, despite all advances in microbiological techniques, there is not truly a valid technique that could serve as a “gold standard” for this [17]. In fact, the accurate diagnosis of PJIs often involves a combination of multiple factors, including symptoms, signs, synovial fluid cell count, serum inflammatory markers, and culture. Culture is still an important diagnostic tool for PJIs [6]. Of equal concern is the fact that antibiotics administered systemically are shown to have a lesser efficacy and resistance against implant-associated biofilms [17].

Microbiological studies agree that the most frequent causative agent of PJIs is *Staphylococcus aureus*, followed by coagulase-negative staphylococci (CNS), which are involved in 50–60% of PJIs [7,18]. In particular, methicillin-sensitive *S. aureus* (MSSA), CNS, and methicillin-resistant *S. aureus* (MRSA) have percentages of about 35, 23, and 15%, respectively. The most frequently identified CNS species is *Staphylococcus epidermidis* (67.8%) [1,8,9,19,20]. Other Gram-positive bacteria (*Enterococcus* spp., *Streptococcus* spp., *Clostridium difficile*) account for 11.1%, and Gram-negative bacteria (*Pseudomonas aeruginosa*, Enterobacteriaceae, *Proteus* spp.) account for 2.5% of total infections [8,20]. Hence, as a general guide, the antibiotic choice should at least provide adequate coverage against *S. aureus* (including MRSA), CNS, and aerobic Gram-negative bacilli [6].

Frequently, the diagnosis of infection is complicated by the failure of traditional microbiological culture techniques to isolate the causative pathogen, which is due to the fact that microorganisms are organized and embedded within complex structures, namely biofilms, that typically occur on prosthetic surfaces [3,20,21]. The occurrence of biofilm results in difficulties for diagnosis and treatment: the adhesion of microorganisms to prosthetic surfaces reduces their likelihood of detection, and the microenvironment within the biofilm protects bacteria from antibiotics applied in treatment [1,6,7,17,21]. Thus, as the bacterial pattern remains unknown, epidemiological data on PJIs may be valuable for guiding orthopedic surgeons in selecting the most effective empirical antibiotic treatment to manage infectious pathogens; however, this may result in the development of drug-resistant bacteria. Infection caused by drug-resistant bacteria, a major global public health problem, requires more surgeries, which leads to higher incidences of complications, higher rates of treatment failure, longer hospital stays and often poor clinical efficacy [7,8,14].

To reduce PJI rates, more effective prevention strategies should be adopted prior to any surgical intervention, with particular attention paid to modifiable risk factors; consequently, close cooperation among various specialists, including orthopedic surgeons, infectious disease experts, microbiologists, engineers, and pathologists, is needed [1,22]. Biomaterial-based approaches have also shown promise for treating PJIs: improving resistance to biofilm formation on the implant surface reduces the rate of post-PJI treatment failure.

The ultimate goal of this research is the design and development of novel poly(ε-caprolactone) (PCL)-based biomaterials for use in tissue engineering. PCL, along with polyhydroxyalcanoates, is the most widely diffused biodegradable and non-cytotoxic polymer, used in many FDA-approved surgical implants and in drug delivery devices for regenerative medicine. Interest in this synthetic polymer arises from its rubbery property, adjustable biodegradability, ease of preparation of blends, composites and co-polymers, which make it a suitable material for soft and hard tissue engineering, for surgical sutures, and for drug delivery vesicles [23,24,25].

In contrast to previous publications, where electrospinning was used to produce PCL scaffolds mainly as 2-dimensional (2D) fibrous structures [26], in this work, highly porous 3-dimensional (3D) constructs were fabricated using the solvent casting/salt leaching method. In addition, the polymer composition was modified with biphasic calcium phosphate [BCP; a 70:30 weight mixture of hydroxyapatite (HA) and beta tricalcium phosphate (β-TCP)], to impart bioactive/bioresorbable properties [13,14], and with silver nitrate, to provide antibacterial properties against the Gram-positive *S. aureus*, one of the most important bacteria involved in PJIs. Furthermore, to assess their potential outcome in promoting physiological bone integration, preliminary tests were performed on sarcoma osteogenic-2 (Saos-2) cells to assess cell vitality and proliferation.

## 2. Results

### 2.1. PCL-Based Biomaterial Morphological and Chemical Characterization

As reported in detail in Table 1, all the prepared PCL-based biomaterials had a cylindrical geometry and similar dimensions, namely diameter (mm) and height (mm), independent of the porous agent used (sodium chloride, NaCl, A and sodium nitrate, NaNO_3_, B), whereas the BCP/PCL materials showed a higher density with respect to the pure PCL samples (*p* < 0.0001). An average density of 0.14 ± 0.02 g/cm^3^ characterized the porous PCL samples, which was slightly lower than the theoretical value (0.20 g/cm^3^) calculated on the grounds of the former pore volume (~83 vol%) in the mixture. In the case of the BCP/PCL scaffolds, the average density increased to 0.23 ± 0.01 g/cm^3^ (Table 1), but additionally, the value was significantly lower than the nominal value (0.32 g/cm^3^) for a ~80% porous material. This means that, besides the macroporosities created by the dissolution of salt granules, additional (micro)porosity was found, reasonably imputed to acetone evaporation, as already demonstrated in the literature [27]. Neither the addition of silver nor salt significantly affected the density values (Table 1).

**Figure 1:**
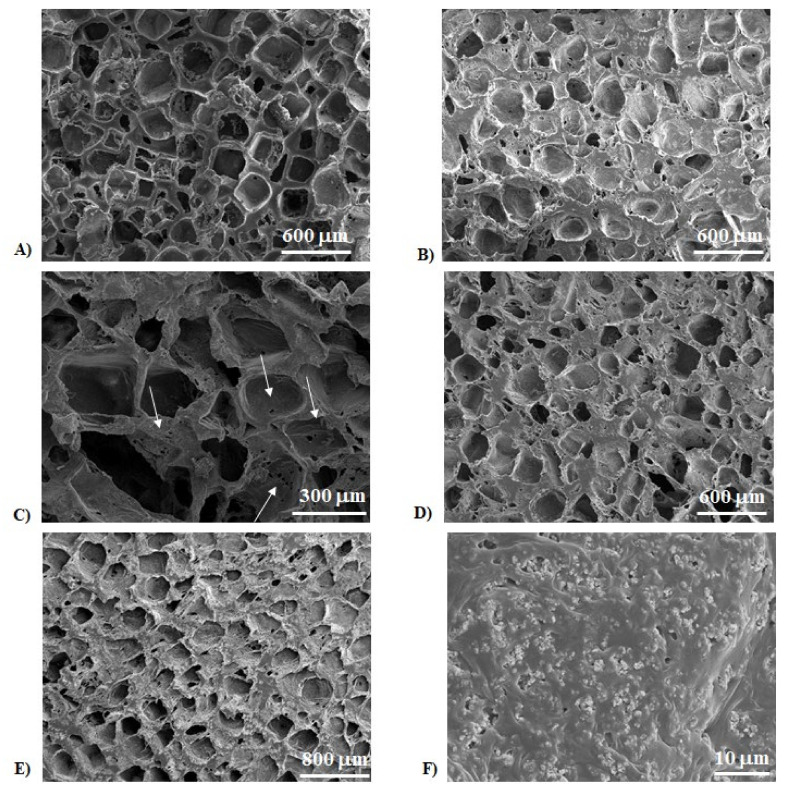
Field emission scanning electron microscopy (FESEM) micrographs of poly(ε-caprolactone) (PCL) scaffolds obtained by using sodium chloride, NaCl (**A**) and sodium nitrate, NaNO_3_ (**B**) salts as templates (analysis carried out on the material sections); (**C**) higher magnification FESEM micrograph of a sample obtained by using NaCl, showing microporosities within the struts, and on the pore walls; (**D**) micrograph of PCL + silver (Ag), showing the same morphology of the neat polymer scaffold; (**E**) lower and (**F**) higher magnification micrographs of a biphasic calcium phosphate (BCP)/PCL sample showing the fine and homogeneous distribution of the calcium phosphate particles inside the polymer matrix.

Figure 1 depicts some representative field emission scanning electron microscopy (FESEM) micrographs of the sections of porous PCL samples, obtained by using both NaCl (Figure 1A) and NaNO_3_ (Figure 1B) pore formers. In each case, the structure was characterized by pores, homogeneously distributed in the materials, the size and shape of which matched well with the templates used (see Figure 13 in the Material and Methods section). In Figure 1C, a higher magnification FESEM image shows the presence of micropores within the struts and the pore walls (white arrows), thus creating an open and interconnected porosity. In addition, from these images, we can observe the presence of thin struts, with a thickness in the range 50–100 μm. In Figure 1D the morphology of the silver (Ag)-functionalized material is depicted: geometrical pores due to salt leaching process, and a high interconnected porosity can be easily observed, suggesting that the silver addition did not affect the scaffold morphology. The same scaffold architecture can be observed when ceramic particles are added to the polymer matrix, as shown in Figure 1E; however, at higher magnification (Figure 1F), calcium phosphate particles can be detected, too. These particles, characterized by sub-micrometric size, formed agglomerates of a few microns, and were homogeneously distributed within the polymer matrix.

**Figure 2:**
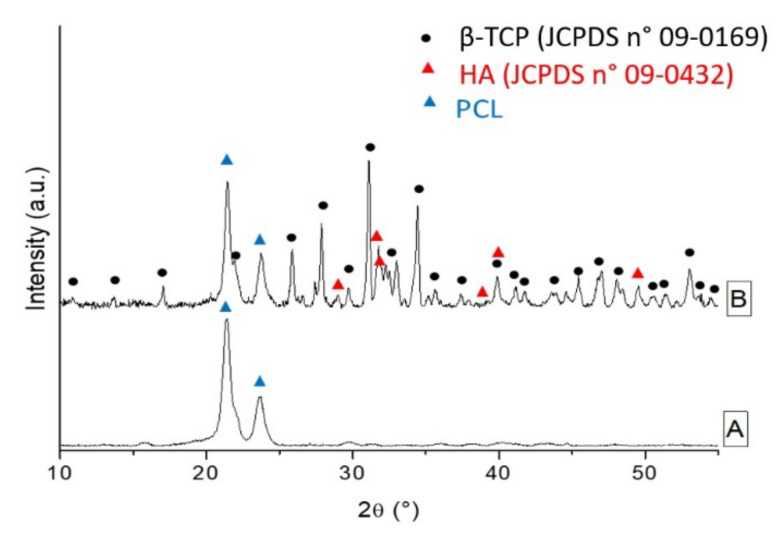
X-ray Diffraction (XRD) patterns of a neat PCL scaffold (**A**) and of the BCP/PCL composite scaffold (**B**).

X-ray Diffraction (XRD) analysis of neat PCL scaffold (Figure 2, curve A) shows the main diffraction patterns of polycaprolactone, precisely the (110) plane at 2θ ~21.4° and the (200) plane at 2θ ~23.7°, suggesting a semi-crystalline nature of the polymer [28,29]. The XRD pattern of the BCP/PCL composite scaffold (Figure 2, curve B) showed the presence of the main hydroxyapatite (HA) and beta tricalcium phosphate (β-TCP) peaks besides those of PCL.

In the case of Ag-functionalized biomaterials, no additional peaks in the XRD spectra were detected due to the low amount of AgNO_3_ added. For these samples, the presence of Ag was confirmed by energy-dispersive X-ray spectroscopy (EDS) analysis. In the case of the scaffolds prepared with a NaCl pore former, Ag concentration ranged between 0.6 and 0.9 wt%, with the highest value corresponding to darker material surfaces, which was in a good agreement with the nominal Ag concentration. According to inductively coupled plasma-mass spectrometry (ICP-MS) analysis, the ionic silver release in the broth corresponded to about 0.15–0.3% of the initial Ag content in the samples where NaCl pore former was added, and in the range 0.7–0.8% for the samples where NaNO_3_ was added. 

**Figure 3:**
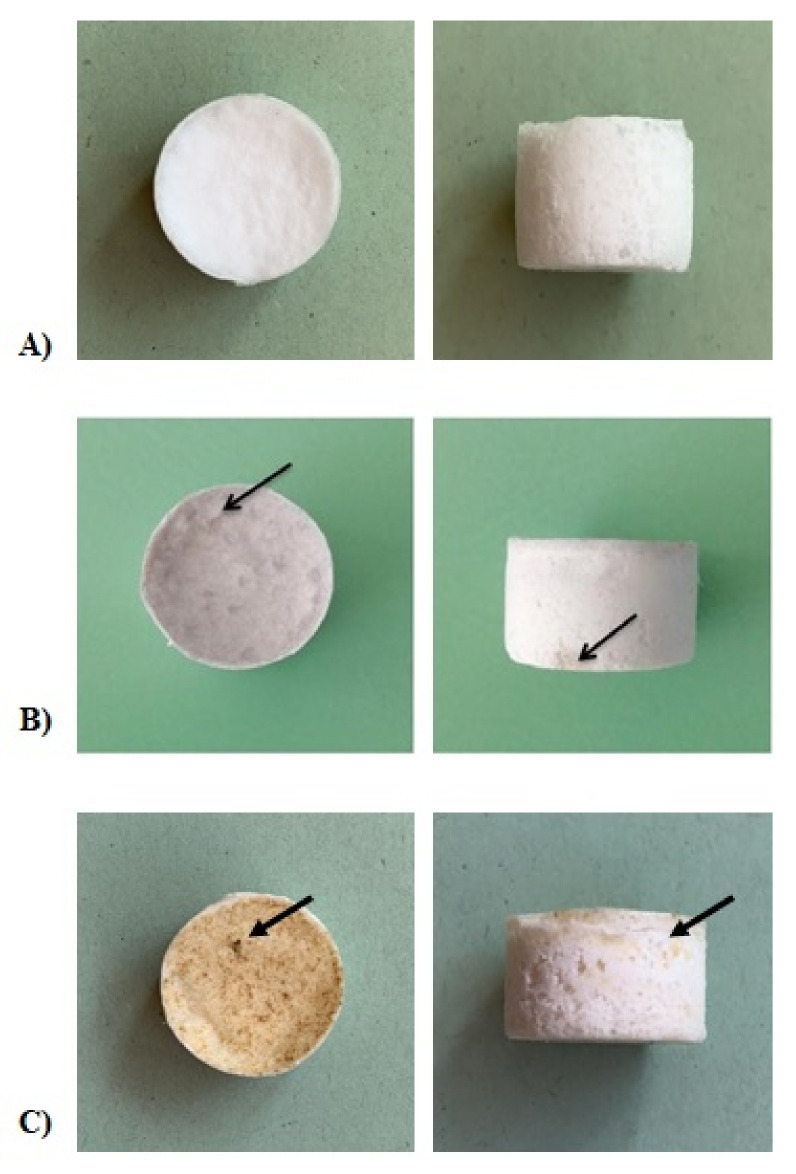
Representative images of PCL-based scaffolds. In detail, BCP/PCL samples with NaCl (**A**); Ag-functionalized, BCP/PCL + Ag samples with NaCl (**B**) and NaNO_3_ (**C**). The Ag-added samples are visibly darker in the images, and the arrows indicate darker spots in which the Ag has formed agglomerations.

Furthermore, as revealed in Figure 3, the presence of silver was also confirmed by the darker color of the Ag-added samples with respect to the non-functionalized samples, which showed a white color. 

**Figure 4:**
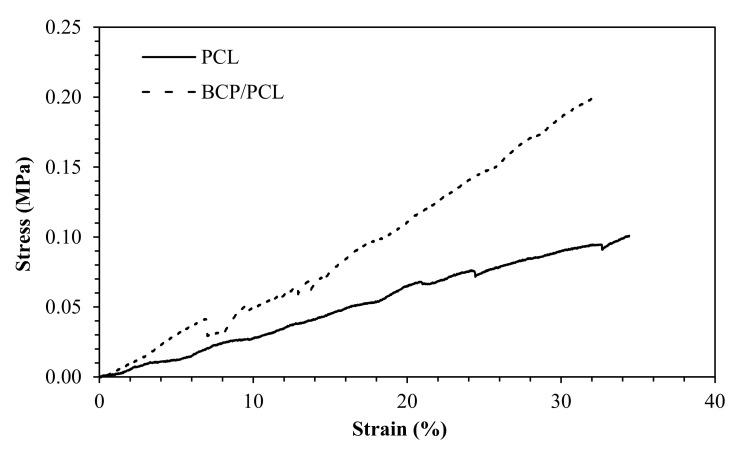
Stress-strain compressive curves of the porous scaffolds: neat PCL (solid line) and BCP/PCL (dotted line) materials.

In Figure 4, the compressive curves of porous scaffolds made by PCL and BCP/PCL are depicted, allowing us to determine a compressive modulus of 0.31 ± 0.02 MPa for PCL, and 0.53 ± 0.07 MPa for the composite material. The results clearly show an important role of the ceramic particles in increasing the stiffness of the porous samples, in agreement with the previous literature [30].

### 2.2. Antibacterial Assay

**Figure 5:**
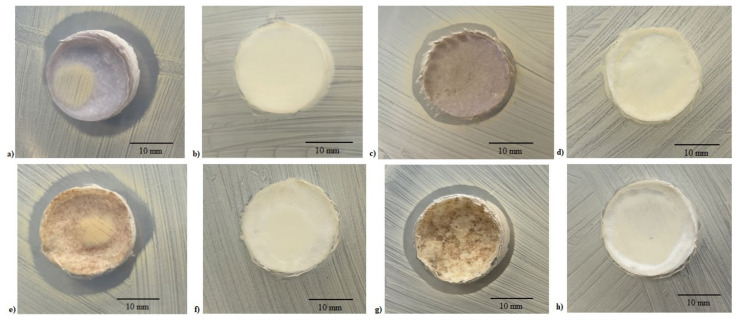
Representative images of the antibacterial tests on the PCL-based samples with NaCl [PCL + Ag, (**a**); PCL, (**b**); BCP/PCL + Ag, (**c**); BCP/PCL, (**d**)] and NaNO_3_ [PCL + Ag, (**e**); PCL, (**f**); BCP/PCL + Ag, (**g**); BCP/PCL, (**h**)] performed by the inhibition halo.

The inhibition halo assay further demonstrated the release of silver from the PCL-based Ag-added samples, both NaCl (Figure 5a) and NaNO_3_ (Figure 5e), and it was accompanied by a halo of staphylococcal growth inhibition near the scaffolds with an inhibition diameter of ~25 mm. Similar results were obtained for BCP/PCL + Ag samples (NaCl: Figure 5c; NaNO_3_: Figure 5g), whereas no inhibition halo was observed in samples prepared without silver addition (NaCl: Figure 5b,d; NaNO_3_: Figure 5f,h).

**Figure 6:**
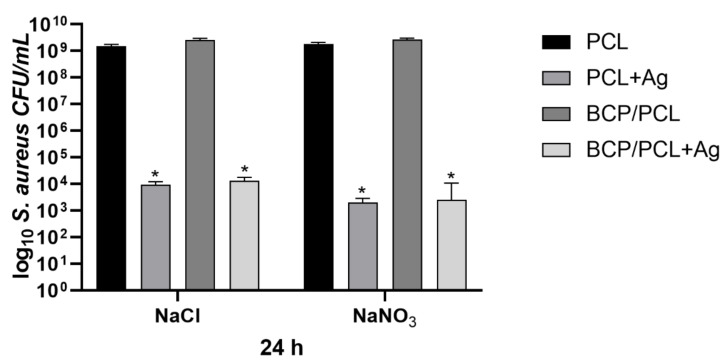
Number of adherent staphylococci (log_10_ colony forming units, CFU/mL) on the scaffolds, produced with either NaCl and NaNO_3_ salts, after 24 h of incubation. Results are the mean values ± standard error of the mean (SEM) of at least three independent experiments. * *p* < 0.0001 unpaired *t*-test.

Figure 6 summarizes the results obtained by the *S aureus* adhesion assays, reporting the number of the adherent staphylococci (log10 colony forming units, CFU/mL) on the scaffold, produced with either NaCl or NaNO_3_ salts, after 24 h of incubation. Specifically, a similar staphylococcal adhesion on the control materials (using the NaCl salt) that were pure PCL and BCP/PCL was reported with values of 1.52 × 10^9^ ± 2.43 × 10^8^ CFU/mL and 2.59×10^9^ ± 3.43×10^8^ CFU/mL, respectively. Whereas a significant (*p* < 0.0001) reduction in bacterial adhesion was obtained on Ag-doped scaffolds, particularly 9.34 × 10^3^ ± 2.70 × 10^3^ CFU/mL for the PCL + Ag and 1.30 × 10^4^ ± 4.45 × 10^3^ CFU/mL for the BCP/PCL + Ag biomaterials (Figure 6). A similar adhesion trend was observed for the scaffolds prepared with NaNO_3_ as a porous agent, with values of 6.41 × 10^3^ ± 1.73 × 10^3^ CFU/mL and 4.44 × 10^3^ ± 1.90 × 10^3^ CFU/mL for the PCL + Ag and BCP/PCL + Ag scaffolds, respectively (Figure 6). 

**Figure 7:**
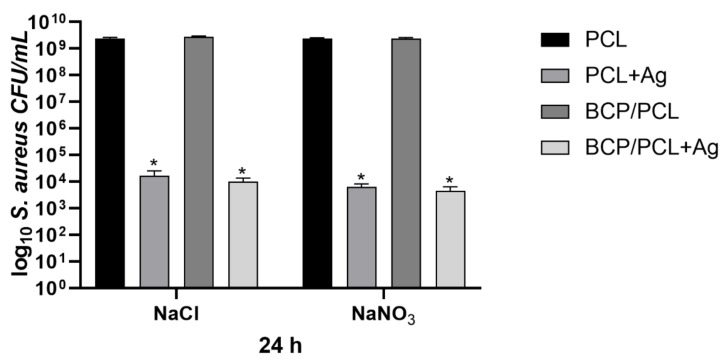
Number of planktonic staphylococci (log_10_ CFU/mL) in the presence of scaffolds, produced with either NaCl or NaNO_3_ salts, after 24 h of incubation. Results are the mean values ± SEM of at least three independent experiments; * *p* < 0.0001 unpaired *t*-test.

The quantification of planktonic bacteria (i.e., those that did not adhere and remained alive in the broth after 24 h of incubation with the scaffold) was conducted to highlight either the role of the scaffold composition itself, potential toxic behavior, or Ag^+^ ion release from the scaffold (Figure 7). Once again, the Ag-free control samples showed a high growth of planktonic bacteria (about 10^9^ CFU/mL, for both NaCl and NaNO_3_). This rate was significantly (*p* < 0.0001) decreased for the Ag-functionalized samples, with values of 1.62 × 10^4^ ± 8.96 × 10^3^ CFU/mL and 6.41 × 10^3^ ± 1.73 × 10^3^ CFU/mL for the PCL + Ag prepared with NaCl and NaNO_3_ salts, respectively; and of 9.91 × 10^3^ ± 3.51 × 10^3^ CFU/mL (NaCl) and 4.44 × 10^3^ ± 1.90 × 10^3^ CFU/mL (NaNO_3_) for the BCP/PCL + Ag biomaterials (Figure 7). Even if comparable, a slightly higher reduction in both adherent and planktonic bacteria was observed in the case of NaNO_3_-added samples, which is in agreement with the higher Ag released in the broth for these specimens. Therefore, the adhesion assay results, together with the inhibition halo data, validated the release of antibacterial Ag^+^ ions from the scaffolds to the surrounding medium as an effective tool for eradicating bacterial contamination at the surgery site.

**Figure 8:**
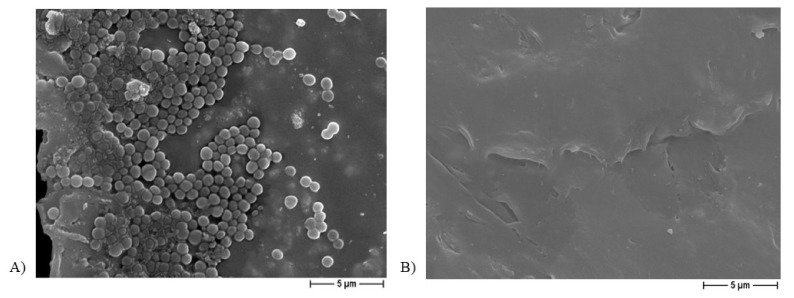
Representative FESEM micrograph of non-sonicated PCL-based scaffolds (**A**) and Ag-added PCL-based scaffolds (**B**), obtained by using NaCl salt as a template, at 5000× magnification.

Figure 8 shows a representative image from the FESEM analysis of a PCL-based biomaterial after 24 h of incubation with the bacterium, but not sonicated, additionally demonstrating the presence of *S. aureus* clusters (Figure 8A), whereas no bacterial aggregates were observed on the Ag-functionalized scaffolds after the incubation period (Figure 8B).

### 2.3. In Vitro Saos-2 Cell Viability/Proliferation Assay

To assess the potential influence of both PCL-based/porous agents and silver on sarcoma osteogenic-2 (Saos-2) cell viability/proliferation, 3-(4,5-Dimethylthiazol-2-yl)-2,5-Diphenyltetrazolium Bromide (MTT) assays and FESEM analysis were performed on PCL-based biomaterials within 14 days of incubation. 

**Figure 9:**
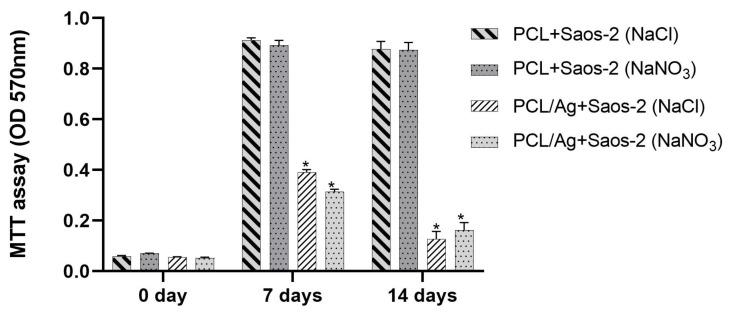
Cell viability (3-(4,5-Dimethylthiazol-2-yl)-2,5-Diphenyltetrazolium Bromide, MTT analysis) of sarcoma osteogenic-2 (Saos-2) cells exposed to PCL-based biomaterial fabricated with NaCl or NaNO_3_ salts and Ag-enriched, expressed an optical density (OD) at 570 nm. Results are the mean values ± SEM of at least three independent experiments; * *p* < 0.0001 unpaired *t*-test.

The effect of NaCl/NaNO_3_ in the presence or absence of silver on Saos-2 cell viability, evaluated as optical density (OD 570 nm), is shown in Figure 9. In particular, at the beginning of incubation (0 day), a similar OD was measured for all the PCL-based scaffolds, with no differences between the controls and silver-enriched biomaterials. No cytotoxic effect was observed after 7 and 14 days of incubation, since the OD values measured for PCL-based biomaterial using NaCl or NaNO_3_ salts were similar, whereas the addition of silver on both scaffold types resulted in a significant (*p* < 0.0001) reduction in Saos-2 cell viability, with OD values halved compared to controls at 7 days, and further reduced at 14 days of incubation. Moreover, for PCL-based scaffolds, the addition of either NaCl or NaNO_3_ stimulated cell proliferation after 7 days with Saos-2 cells, reaching a state of confluence with no further increase at 14 days, whereas the presence of silver slightly supported the growth of cells, but only after 7 days (Figure 9). 

**Figure 10:**
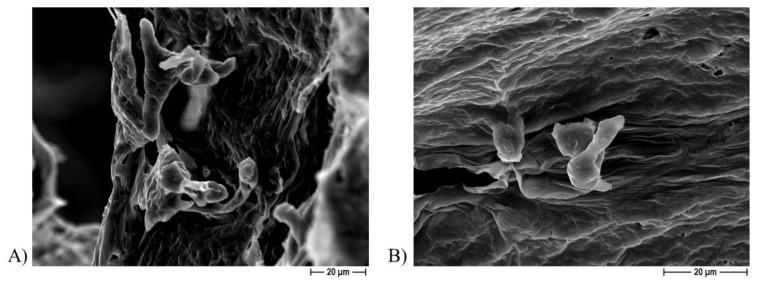
Representative FESEM micrographs of PCL scaffolds obtained by using NaCl salt as template showing presence of Saos-2 cells present at 1000× magnification after 7 days (**A**) and at 1500× magnification after 14 days (**B**) of incubation.

**Figure 11:**
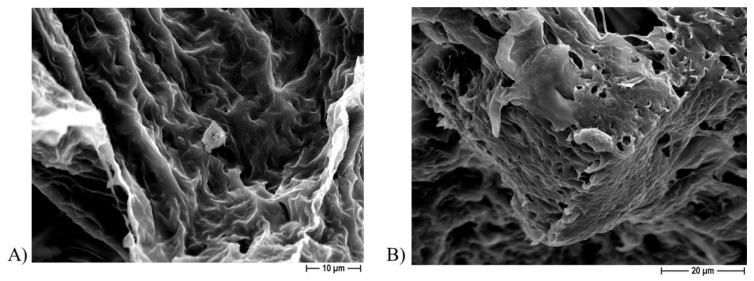
Representative FESEM micrographs of PCL scaffolds obtained by using NaNO_3_ salt as a template showing presence of Saos-2 cells at 1000× magnification after 7 days (**A**) and at 1500× magnification after 14 days (**B**) of incubation.

FESEM analysis revealed the presence of osteoblast-like cells on the PCL-based scaffolds obtained by using either NaCl (Figure 10) or NaNO_3_ (Figure 11) as a template within 7 and 14 days of incubation, thus confirming that cells can attach to the biomaterial independently on the porous agent. 

When BCP was added to the PCL-based scaffolds, the viability results obtained by MTT were similar to those indicated by the OD values, and no difference was noted in the FESEM observation either (data not shown).

Altogether, MTT and FESEM analyses indicated that the addition of silver at a concentration of 1.06% (by weight with respect to PCL) to the scaffolds was considerably high for cell survival, thereby determining a relevant reduction in Saos-2 cell viability and proliferation on all the tested biomaterials starting from 7 days of incubation.

## 3. Discussion

In surgery, device implantation results in a non-negligible occurrence of infections, one of the major causes of morbidity and mortality in this medical field [3,31]. A foreign body is the triggering event for biomaterial-associated infections (BAIs) because certain features of a biomaterial, such as its surface and roughness characteristics, not only attract host eukaryotic cells involved in tissue regeneration, but also free-floating bacteria. Indeed, as soon as a contamination occurs, the “race to the surface” takes place, followed by microorganism adhesion and subsequent biofilm formation, which are important in determining the occurrence of infection [7,17,32]. 

These concerns led to the exploration of synthetic biodegradable polymers, such as PCL, a hydrophobic bioresorbable material frequently proposed for tissue engineering, for use either as a porous scaffold in bone tissue reconstruction or as a vehicle for the controlled delivery of therapeutic molecules [20,33,34,35,36,37,38].

The manipulation of biomaterial surface properties to control the interaction between implants and their biological surroundings has been one of the major research topics in the biomaterials field [3,7,39,40]. In fact, the incorporation of stiff, bioactive inorganic materials [e.g., calcium phosphates (CaPs) such as HA and β-TCP] into a PCL polymer leads to significant enhancements in mechanical properties, bioactivity, and in vivo bone regeneration capacity [36,41]. HA is the predominant crystalline form of CaPs, constituting 60–70% of bone tissue and providing mechanical strength. β-TCP is highly biocompatible and creates a resorbable interlocking network within the defect site to promote healing [42,43]. CaP compounds have emerged as prominent materials for biomedical applications, and are mainly used as bone substitutes due to their desirable non-cytotoxic, non-inflammatory and non-immunogenic properties. They also bond directly to new bone without requiring intermediate connective tissue [3,44]. Owing to these features, both HA and β-TCP and their mixtures, denoted as BCP, are commonly used as fillers in polymer-based bone substitutes. Thus, when implanted in an osseous site, bone bioactive materials provide an ideal environment for cell adhesion and colonization with high osteoconductivity (supporting bone growth and encouraging the ingrowth of surrounding bone), as well as osteoinductivity (promoting the differentiation of progenitor cells in the osteoblastic lineage) [3,39,44].

Hence, the modification of the biomaterial surface is aimed not only at improving the final biocompatibility or the cell-interactive behavior at the implant–host interface but also at preventing and controlling implant infections by inhibiting microbial adhesion to device surfaces [3,20,38,45]. To face the problem of microbial contamination and to prevent the development of PJIs, the proposed solution is antibiotic release from the biomaterial, but it is important to appreciate that this treatment also contributes to antimicrobial resistance. Therefore, an efficient alternative to conventional methods of overcoming the growing problem of this resistance would be to incorporate inorganic antibacterial agents (such as silver, copper, or zinc ions) or natural antimicrobial compounds (chitosan, carbon-derived components) into prosthetic materials to endow them with broad antimicrobial activity against Gram-negative and Gram-positive bacteria and fungi [17,20,38,39,45,46,47]. This has been the subject of extensive research, but the desirable successful prevention or effective solution is yet to be achieved.

On this basis, the aim of the present research is the design and characterization of 3D PCL-based scaffolds either functionalized with BCP or enriched with silver to assess their antimicrobial efficacy against *S. aureus*, and to evaluate their ability to promote osteoblast-like cell vitality and colonization.

Biodegradable polymers, such as PCL, have emerged as synthetic grafting materials, and have been used as a valuable alternative to autografts and allografts [48]. PCL shows excellent biocompatibility, proper biodegradability, high toughness, and good mechanical resistance [49], which is why it is the synthetic polymer most commonly used for biomedical applications [50]. Moreover, when bioactive ceramic particles, such as CaPs, are added to the polymer matrix, better bone interactions and improved cell adhesion with the biological environment is observed [50]. 

The technology most commonly used to fabricate PCL biomaterials is electrospinning, a flexible technique that produces fibers with diameters ranging from a few micrometers to nanometers [50]. Typically, this process leads to the fabrication of membranes or 2D structures, while bone regeneration requires the use of a 3D construct of a certain thickness to fill sometimes large defects and to interact with the surrounding tissue. Therefore, 3D porous scaffolds (~1 cm high) were developed by a simple and versatile technology that offers several advantages. First, solvent casting, which consists of the dissolution of PCL pellets into acetone and the pouring of the solution into molds, allows the final material to be easily shaped into the form and dimensions required by the clinicians. Acetone is an appropriate dispersion medium for fine HA/β-TCP particles and the silver precursor because it favors the homogeneous distribution of both CaPs and Ag into the polymer matrix. Finally, the salt leaching method was successful in producing the required porosity in which the pore size can be adjusted according to the particle size distribution of the salt. As a result, porous scaffolds (either made by pure PCL or BCP/PCL composite), characterized by a high degree of porosity (>80%) and interconnected pores as required by bone regeneration applications [51], were successfully fabricated.

The microbiological results achieved in the current work demonstrate the strong anti-adhesive and anti-staphylococcal activity of the silver-enriched PCL biomaterials (*p* < 0.0001). In fact, a significant reduction in the adherence of *S. aureus* onto Ag-doped materials—independent of the salt used as pore former—was obtained, with values of about 10^3^–10^4^ CFU/mL compared to the controls (10^9^ CFU/mL). The BCP presence did not interfere with the anti-adhesive features. A comparably significant (*p* < 0.0001) reduction trend was also recorded for planktonic bacteria, with a decrease in bacteria count of about 6 log: 10^9^ CFU/mL of PCL-based scaffolds vs. 10^3^ CFU/mL of Ag-doped PCL-based biomaterials. Of note, a slightly higher reduction in both adherent and planktonic *S. aureus* was observed in the case of NaNO_3_-added samples, which is in agreement with the higher Ag released in the broth for these specimens. Why such relevant effect on staphylococcal growth inhibition? We speculate that different silver activities might perform different functions: before biofilm formation, Ag^+^ directly killed *S. aureus* in planktonic form, but thereafter, its action was directed at adhering staphylococci during the early stages of biofilm development, thereby preventing complete biofilm formation. The latter activity was also supported by FESEM analysis of the Ag-added PCL scaffolds, in which no biofilm presence was observed on the non-sonicated biomaterials after 24 h of incubation with *S. aureus*. Therefore, the adhesion assay results, together with the inhibition halo data, validate the strategy of antibacterial Ag^+^ release from the Ag-enriched PCL-scaffolds into the surrounding medium as an effective tool for eradicating bacterial contamination from the surgery site, further confirming the anti-staphylococcal action of Ag^+^ [17,52]. To date, Ag^+^ has demonstrated antibacterial efficacy, since biomolecule components of proteins, enzymes, and cell-membrane generally contains active groups capable of coordinating with silver cations (Ag^+^) leading to bacterial cell death [17]. Most of the destructive pathways in bacterial cells utilize the generation of reactive oxygen species (ROS); hence, it is known to be the most potent component behind bacterial cell death [52,53]. 

In the literature, microbiological data on 3D PCL-based biomaterials are still rare; however, principal results on PCL fibers/membranes for skin tissue engineering have been reported [20,38,40,45,54,55]. The first mention is in a report of the antibacterial activity of 3D-printed silver-doped PCL–propylene succinate films, where notable antibacterial behavior against several microorganisms (*S. aureus*, *Escherichia coli*, *Ps. aeruginosa*, and *Candida albicans*) was revealed simply by the presence of an inhibition halo [40]. Tardajos M. and co-authors prepared both electrospun PCL membranes and 3D-printed PCL scaffolds, functionalized with chitosan, to assess their antibacterial features. For the 3D printed biomaterials, this also involved the demonstration of a significant reduction in *S. aureus* and *S. epidermidis* CFU/mL after 24 h of incubation [45]. In addition, similar results were obtained from another research group that evaluated 3D-printed PCL biomaterials incorporated with graphene, which boosted the bactericidal effect of the doped materials towards *S. epidermidis* and *E. coli* [20]. Motealleh et al. designed drug-loaded electrospun PCL nanofibers and demonstrated a good inhibition of *S. aureus* and *C. albicans* growth in the presence of chamomile [54]. Other authors reported that polyvinylpyrrolidone–iodine PCL films significantly decreased *E. coli* growth [55]. In addition, we compared our data with those of Cara et al., who prepared bone cement samples loaded with antibacterial agents (i.e., vancomycin, clindamycin, and gentamicin) and demonstrated relevant anti-staphylococcal activity [7]; these results, obtained with the use of antibiotics, were very close to ours achieved with silver. 

The silver functionalization of the PCL-based scaffolds had no impact on their morphological features, as confirmed by the sample FESEM analysis. The amount of AgNO_3_ added to the PCL and BCP/PCL porous materials was performed according to the literature, in which collagen/PCL porous materials were loaded with Ag nanoparticles in the range of 0.5 to 2 wt% (showing the best antibacterial properties at 2 wt%), while lower contents also resulted in good cell proliferation and growth [56]. Dobrzański et al. [57] prepared polycaprolactone fibers with 0–5 wt% of AgNO_3_, and it showed high antimicrobial activity against *S. aureus* even at a low concentration (1 wt%, 99.9% efficacy), that further was increased by the addition of AgNO_3_.

**Figure 12:**
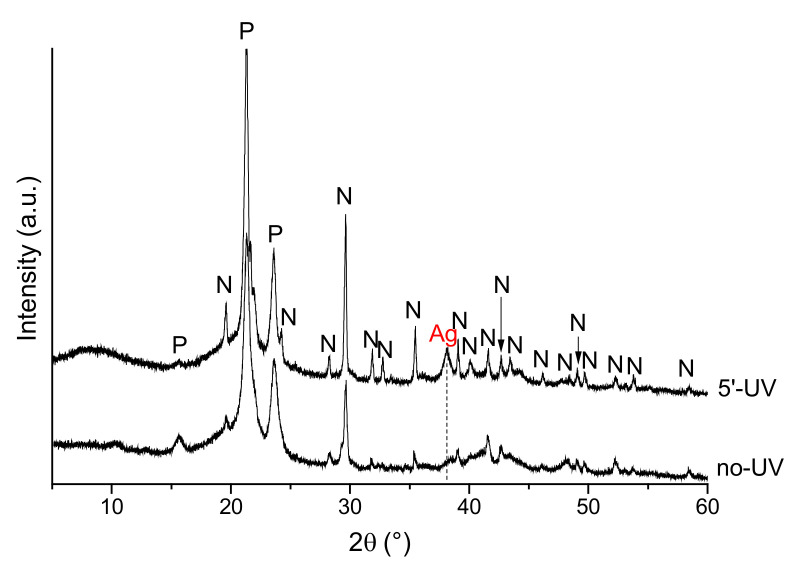
XRD patterns of silver nitrate (AgNO_3_)-PCL samples before and after ultraviolet (UV) irradiation (P = PCL; N = AgNO_3_; Ag = metal silver).

It is well known that Ag compounds, such as AgNO_3_ and silver halides, show a high photosensitivity under visible and ultraviolet (UV) light [58,59], which corresponds to the chemical reduction of Ag^+^ ions to metallic Ag nanoparticles [60]. To verify this effect, XRD analysis was performed on a PCL–silver nitrate (AgNO_3_) sample before and after UV irradiation for 5 min (Figure 12). This sample was prepared as a solid material, without any salt addition to create the macroporosity. In such a way, the silver concentrated onto the surface and in small volume of the polymer samples, thus to be detected by XRD. The formation of metallic silver after UV irradiation is clearly observable, while small signals, in the form of traces, seem to be already present in the non-irradiated samples, thus confirming the possible Ag reduction under visible light. 

This photosensitivity produced a change in sample color. In the materials developed here, a role also seemed to be played by the applied porogen. In fact, the samples with added NaCl showed a progressive surface darkening, while those prepared with NaNO_3_ turned yellow–brown after light exposure (Figure 3). In the former case, an ion exchange between NaCl and AgNO_3_ salts was postulated to have occurred during the washing to leach out the NaCl granules and produce the porous constructs. Thus, it is reasonable to assume that while the samples prepared with NaNO_3_ contained pure AgNO_3_, in those fabricated with NaCl, surface AgCl was also contained even if not detected by XRD analysis. This difference in surface chemistry between the two sample types produced a slightly different antimicrobial behavior in the count of both adherent and planktonic staphylococci after 24 h of incubation. Finally, XRD results presented in Figure 12 show that silver is predominantly contained in an ionic form in the scaffold, even if a partial reduction to metal Ag can occur under light exposition, as already observed in the literature [61]. Such findings strengthen the antibacterial mechanism of Ag^+^ species, as previously discussed.

The elastic modulus of PCL porous scaffolds, determined under compressive tests, provided a value of 0.31 ± 0.02 MPa, this value being almost doubled (0.53 ± 0.02 MPa) when BCP particles are added to the polymer, showing the remarkable effect of the ceramic filler in improving the stiffness of the scaffolds. Although the increase in the compressive modulus due to the addition of CaP particles is well documented in the literature [30,62,63], a comparison between these mechanical data and those present in the literature is particularly hard. This is due to the many differences, in terms of porosity of the scaffolds (both porosity amount and size), structure and degree of crystallinity of the polymer, ceramic filler amount, geometry and dimension of the specimens, and overall manufacturing methodology. From a literature survey, it can be easily observed that most 3D scaffolds are prepared by printing technologies (such as selective laser sintering [64], fused deposition modeling [62,65], solid free form fabrication [30]) and are characterized by lattice structures made by aligned filaments, with compressive moduli typically of the order of a few tens of MPa. Although in some cases the porosity amount and the size of the macropores is comparable with that of the scaffold objects of this research, it is observed that the filaments (therefore, the cell wall) typically have a thickness of a few hundred microns, while the materials prepared by salt leaching show thin struts of about 50–100 μm, as already reported in the Results section, partially explaining the difference in stiffness. Another preparation method implies the fabrication of scaffolds by freeze casting [63] or extrusion followed by a freeze-casting process [66], providing scaffolds of significantly lower stiffness. For instance, Choi et al. [63] determined a compressive moduli from 0.1 MPa (neat PCL) to 2.7 MPa, while increasing the HA content; however, the porosity was micrometric, and the pore size decreased by increasing the HA content. Finally, it is observed that the modulus values were in a good agreement (or even higher) with those of the literature, when the scaffolds are prepared with the same technology. Chuenjitkuntaworn et al. [67] produced PCL scaffolds for bone tissue engineering through a solvent casting/particle leaching method (using sucrose instead of the salt) and determining a compression modulus from 0.13 MPa (neat PCL) to 0.32 MPa for a 50% HA-added polymer. As a general comment, it seems that the adopted technology leads to relatively low mechanical properties for the scaffolds, strengthening their role more as bone filler materials (able to adapt to the defect shape, while providing the correct biological and physical environment for bone healing [68]) or drug delivery vehicles, than as load-bearing implants. At the same time, what emerged from the literature is that higher mechanical properties can be obtained by varying the structure of the scaffolds, for example with a lower porosity or a smaller pore size, which is easily tunable by controlling the quantity and size of the starting porogenic salt, thus to adapt the scaffold to the individual clinical needs. Here, we report on the preparation of PCL-based scaffolds, designed with orthopedic application in mind, and presented preliminary results on Saos-2 cell viability and cell hosting ability. First of all, we demonstrated the non-toxic effect of the PCL itself; in fact, a similar viability was reached within 7 and 14 days of incubation by MTT assays, regardless of the salt used as a pore former during the manufacturing. In addition, FESEM analysis confirmed the presence of osteoblast-like cells on the PCL-based scaffolds. These results are in good agreement with those of other researchers who demonstrated the non-toxic effect of PCL on fibers and human cells, such as fibroblasts [20,40,45], mesenchymal stem cells [54], or osteoblasts [38]. The addition of silver—the release of which was quantified by ICP-MS as 0.15–0.8 wt% of the initial Ag content in the samples—to the biomaterials revealed a relevant reduction in Saos-2 cell viability up to 14 days of incubation. Our findings were in line with those of Binkley et al., who demonstrated the toxic effect of PCL fibers enriched of silver (5 wt%) [69]; furthermore, either Yang et al. or Albers et al. revealed a Saos-2 cell toxic effect of silver-nanoparticles (at high doses) with respect to the controls [70,71]. Why this undesirable effect of silver on human osteoblast-like cells? We hypothesize that these cell can have a greater susceptibility to silver with respect to other human cells [70,71,72,73], which is mainly dose-dependent and attributable to their increased internalization of metal ions [71]. In fact, other works have demonstrated the non-toxic effect of silver (5–20 wt%) in bioglass-based biografts [72], in titanium Ag-coated (about at 1%) materials [73], or in Ag-nanoparticles (0.1–1 mM) [70]. However, all these results are difficult to compare with ours, since different methodologies or biomaterials were used. Hence, it is essential to explore the silver–dose correlation in antimicrobial and cytotoxic effects to evaluate the clinical applicability of silver since very close concentrations could be antibacterial and non-harmful for human cells at the same time. 

Despite the limitations of the present study—mainly that only one representative Gram-positive microorganism was tested and the silver dose used turned out to be not completely biocompatible—the combination of Ag–PCL scaffolds with osteoregenerative calcium phosphates to develop sustainable solutions for PJI prevention and treatment, without resorting to conventional antibiotic therapies, is considered an opportunity with the potential of progressing to more sophisticated platforms to target bone tissue regeneration.

## 4. Materials and Methods

### 4.1. PCL-Based Scaffold Preparation

**Figure 13:**
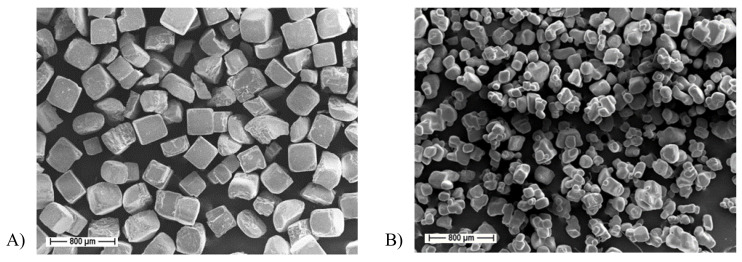
FESEM micrographs of commercial NaCl (**A**) and NaNO_3_ (**B**) salts, used as pore formers to prepare the scaffolds.

PCL (molecular weight 80,000, density of 1.145 g/cm^3^; Sigma Aldrich, Milan, Italy) pellets were dissolved in acetone (acetone–PCL weight ratio 80:20) at 40 °C for 24 h. As pore formers, both NaCl and NaNO_3_, (Sigma Aldrich, Milan, Italy, >99.5% purity, sieved in the range of 125–355 μm) were used. In Figure 13, the FESEM (Zeiss Supra 40, Jena, Germany) micrographs of both salts are depicted. NaCl granules were characterized by regularly shaped grains, most of them cubic, with an average size of about 330 μm. On the other hand, NaNO_3_ granules were characterized by smaller grains (average size of about 270 μm) with more rounded edges.

The salts were added to the polymer solution (NaCl or NaNO_3_–PCL weight ratio 90:10, to produce a microporosity of about 80 vol%) under magnetic stirring, to promote homogenization. The mixture was then cast into cylindrical plastic molds (20 mm diameter, 10 mm height). Samples were dried into closed chambers for 3 days, then demolded and immersed in deionized water for 4 days to leach out the salt crystals. 

For the development of the porous BCP/PCL composites, the BCP mixture was prepared by dry mixing HA (Captal S BM192, batch P270S, Plasma Biotal, Buxton, UK) and β-TCP (Captal R, batch P364S Plasma Biotal, Buxton, UK) powders in a 70:30 weight ratio. 

**Figure 14:**
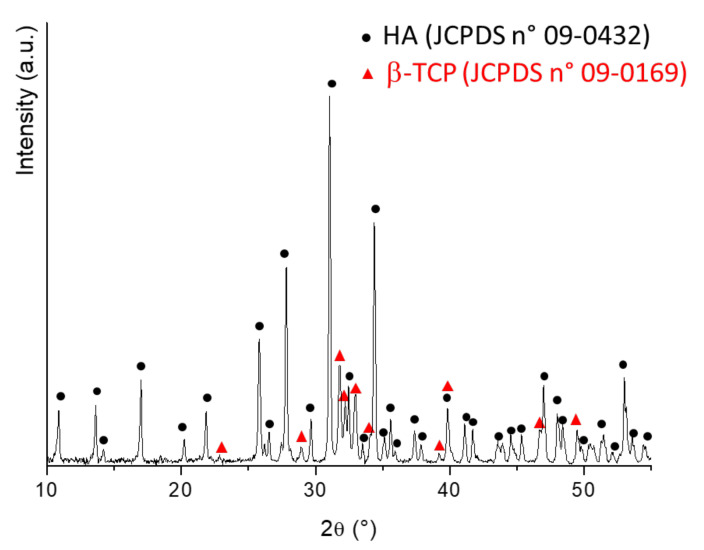
XRD pattern of the BCP mixture, showing that it is purely composed of the HA and β-TCP phases.

In Figure 14, the XRD (Philips PW 1710, Malvern, United Kingdom) analysis of the mixture is reported. All diffraction patterns were indexed according to the Joint Committee Powder Diffraction System (JCPDS) files of HA (09-0432) and β-TCP (09-0169). This mixture was then added to acetone and stirred for 12 h, to achieve a homogeneous suspension. Subsequently, PCL was added to this suspension (BCP–PCL weight ratio 40:60) according to the procedure previously described. Finally, for the elaboration of Ag-doped materials, a fixed amount of AgNO_3_ (Sigma Aldrich, Milan, Italy, Grade AR, 169.87 g/mol; 1.67% by weight with respect to PCL, which corresponded to 1.06% ionic Ag) was solubilized in acetone, under vigorous magnetic stirring for 24 h. After that, BCP powders (when necessary) and PCL were subsequently added, according to the procedures described above. 

Eight different scaffold types were obtained: pure PCL, BCP/PCL, PCL + Ag, and BCP/PCL + Ag with NaCl or NaNO_3_ salt as pore formers.

**Figure 15:**
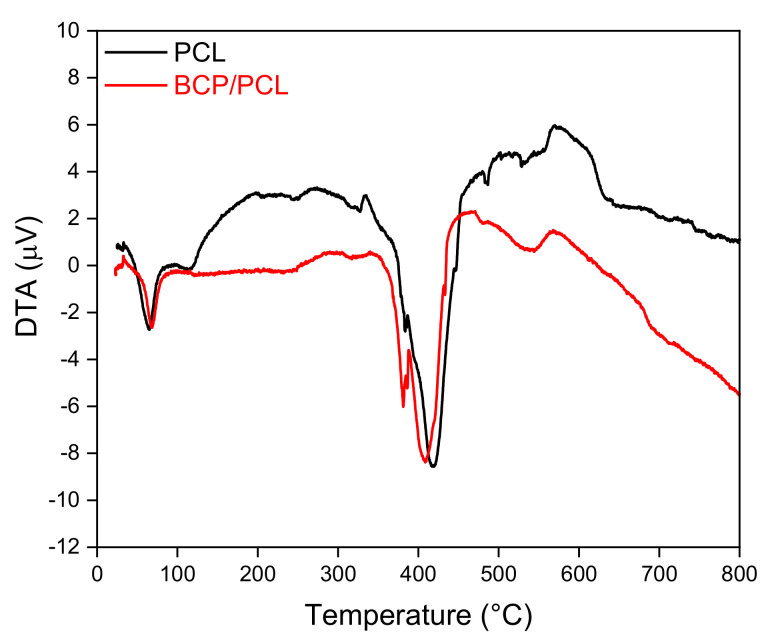
Differential thermal analysis (DTA) curves of PCL and BCP/PCL mixture up to 800 °C (heating rate 10 °C/min).

Differential thermal analysis (DTA, LabSys evo machine, Setaram, Caluire, France, heating up 800 °C at 10 °C/min) was carried out on both PCL and BCP/PCL samples, to evaluate the effect of the ceramic filler on the thermal behavior of the polymer. In Figure 15, the DTA curves of the two materials are depicted. Neat PCL (black line) is characterized by a melting temperature of 65 °C, a strong endothermic decomposition peak at 419 °C and a decomposition exotherm at about 570 °C. It is worth mentioning that such thermal behavior, where the polymer is characterized by a DTA endothermic melting peak, an endothermic decomposition peak and several exothermic decomposition peaks of which the last is normally considered, is characteristic of a well-defined polymer group, of which polycaprolactone is included [74]. Nevertheless, from Figure 15 we can observe that the addition of the ceramic particles (red curve for BCP/PCL) does not significantly affect the thermal behavior, as similar melting (68 °C) and degradation temperatures (409 °C and 566 °C) can be determined, in agreement with previous literature [67]. 

In addition, to investigate the possible silver ion photoreduction, samples simply fabricated by AgNO_3_ added to PCL were prepared. The specimens were irradiated in a UV chamber (Qurtech S.A., model Dynamic Curing Box, Frisange, Luxembourg) and XRD analysis was performed before and after irradiation. 

### 4.2. PCL-Based Scaffold Characterization

The microstructures of the samples were observed using FESEM (Zeiss Supra 40, Jena, Germany), equipped with Oxford EDS microanalysis (Oxford Instrument plc, Abingdon, UK) for chemical analyses. XDR analysis was also performed to investigate the sample chemical composition and chemical state of the elements. The silver ion release from the Ag-doped samples was determined by ICP-MS (Eurolab s.r.l, Nichelino, Turin, Italy). The porous scaffold compressive modulus was determined with a Zwick Roell Z050, universal testing machine (ZwickRoell, Ulm, Germany) using a 50 kN load cell and a crosshead speed of 0.5 mm/min. The load was applied on the top of each scaffold specimen until it was compressed by about 35% of its original height. The compressive modulus was calculated as the slope of the linear portion of the stress–strain curve.

### 4.3. In Vitro Antibacterial Tests

The antibacterial behavior of the silver-added PCL-based scaffolds was investigated by means of the inhibition halo assay in accordance with European Committee on Antimicrobial Susceptibility Testing (EUCAST) guidelines (manual v 9.0; https://www.eucast.org/ast_of_bacteria/disk_diffusion_methodology acceessed on 15 August 2021) and by a bacterial adhesion assay by applying methods used in previous papers of ours [39,46,75,76]. A biofilm-producing *S. aureus* strain (American Type Culture Collection^®^, ATCC^®^ 29213, Manassas, VA, USA) was employed for both the tests because it is one of the main pathogens involved in PJIs.

#### 4.3.1. Inhibition Halo Assay

For the inhibition halo test, which detects the release of silver from treated samples and evaluates its effect on staphylococcal growth, a 0.5 McFarland bacterial suspension, containing approximately 1–2 × 10^8^ CFU/mL, was spread using a sterile swab on Mueller Hinton Agar (MHA, Becton Dickinson and Company, Franklin Lakes, NJ, USA) plates on which the sterile PCL-based samples were placed (with the silver-enriched surface in contact with the plate) and incubated for 24 h at 37 °C. Antibacterial activity was estimated by measuring (mm) the inhibition halo (the zone around the samples where bacterial proliferation stopped), and the antimicrobial efficacy of treated and untreated samples was compared.

#### 4.3.2. Bacterial Adhesion Assay

The antibacterial behavior of the scaffolds was investigated based on a quantitative bacterial adhesion assay, and a sonication protocol was used to dislodge adherent bacteria. Similarly, the effect of different biomaterials on the number of planktonic bacteria was also determined. Specifically, staphylococci stored at −80 °C were cultured overnight at 37 °C in Mueller Hinton Broth (MHB; Becton Dickinson and Company, Franklin Lakes, NJ, USA). After incubation, bacteria were centrifuged, and the pellet was re-suspended in 100 μL of MHB and then diluted in MHB to 10^4^ CFU/mL as confirmed by colony counts on MHA (Becton Dickinson and Company, Franklin Lakes, NJ, USA). The sterile samples were transferred to a 6-well culture plate, covered with 7 mL of staphylococcal suspension, and incubated at 37 °C by shaking for 24 h to allow in vitro bacterial adhesion. Controls represented by bacteria incubated in MHB with no material were also performed. The number of strongly bound bacteria on the scaffolds after incubation was quantified after sonication (40 kHz) for 30 min at 22 °C in 10 mL of sterile saline solution 0.9% (Bieffe Medital S.p.A., Grosotto, Italy). The CFU was quantified in each sonication product by plating the pellet obtained after a centrifugation onto MHA. The number of planktonic bacteria (CFU/mL) was also determined [39]. All experiments, assayed in triplicate, were performed simultaneously for each scaffold type and repeated a minimum of three times. 

### 4.4. Cell Viability Assays by Direct-Contact Test

With orthopedic applications in mind, the human osteosarcoma cell line Saos-2 (ATCC^®^, HTB-85, Manassas, VA, USA), an osteoblastic phenotype, was used for the in vitro experiments. Briefly, Saos-2 cells were cultured in a high-glucose Dulbecco’s modified minimum essential medium (DMEM, Sigma Aldrich, Milan, Italy) with phenol red and supplemented with sodium bicarbonate, 10% foetal bovine serum, and 1% penicillin–streptomycin (pH 7.4), and then incubated at 37 °C in a humidified air atmosphere of 5% CO_2_. After trypsinization, 2 × 10^4^ Saos-2 were seeded onto the surface of sterile scaffolds cut into cylinders (5 mm diameter and 5 mm in height) in 96-well plates and cultured in a 150 μL cell culture medium for different time points (0, 7, and 14 days). All samples were performed in triplicate. The medium was replaced every 2–3 days. Cell viability was assessed at 0, 7, and 14 days by MTT assay (Sigma Aldrich, Milan, Italy). Briefly, the MTT labelling reagent (final concentration 0.5 mg/mL) was added to the wells; samples were incubated for 4 h; then 200 μL of dimethyl sulfoxide (Sigma Aldrich, Milan, Italy) was added for 30 min and OD was measured at 570 nm using a microplate reader (VICTOR3TM, PerkinElmer, MA, USA).

At each time point, data were expressed as the OD considering the OD of the PCL-based scaffolds with Saos-2 cells minus the OD of the PCL-based scaffolds without Saos-2 cells. The Saos-2 cell viability at the same time points was further evaluated by means of FESEM (Zeiss Supra 40, Jena, Germany) analysis. 

### 4.5. Statistical Analysis

The microbiological data, expressed as CFU/mL, and the MTT test data, expressed as OD (570 nm), were analyzed by descriptive statistics (mean values and standard error of the means, SEM) and tested by an unpaired Student’s *t*-test to highlight significant differences (*p <* 0.05) between the different materials using the GraphPad Prism 9 software (San Diego, CA, USA).

## 5. Conclusions

Although biomedical implants have transformed medicine, they have also increase the risk of infection. Implanting any surgical medical device is invasive and triggers an immune reaction to the foreign body, and this condition induces vulnerability to microbial attack, mainly by opportunistic pathogens. 

In the present research, multifunctional PCL scaffolds fabricated by a salt-leaching process were successfully loaded with biphasic calcium phosphates to improve the bio-mechanical/bioresorbable properties of bone regeneration and with silver to determine anti-infection features by discouraging bacterial adhesion. Remarkably, the incorporation of silver into the biomaterials led to noteworthy enhancement of its in vitro antibacterial behavior, on both adhered and planktonic staphylococci. The dual nature of the 3D PCL scaffolds showed great potential for engineering bone tissue and reducing PJIs as a promising microbial anti-adhesive tool used in the delivery of targeted antimicrobial molecules, even if the silver amount needs to be tuned to improve osteointegration. 

The biological characterization of the materials obtained in this research was limited to a preliminary test of antibacterial behavior to verify the achievement of the target. A more detailed biological investigation of the most promising surface is currently ongoing and will be published in further work.

## Figures and Tables

**Table 1:** Morphological characteristics (reported as mean ± standard deviation) of the different PCL-based scaffolds obtained with both NaCl (**A**) and NaNO_3_ (**B**).

A		Morphological Parameters				Statistical Analysis
		Diameter (mm)	Height (mm)	Weight (mg)	Density (mg/mm^3^)	Student’s *t*-test
Scaffold Type	Porous Agent					
PCL	NaCl	17.62 ± 0.03	9.03 ± 0.59	311.2 ± 21.23	0.14 ± 0.02	weight and density PCL vs. BCP/PCL *p* < 0.0001
BCP/PCL	NaCl	17.93 ± 0.12	9.53 ± 0.77	566.2 ± 32.32	0.23 ± 0.01	
PCL + Ag	NaCl	18.46 ± 0.23	10.04 ± 0.52	468.6 ± 12.15	0.17 ± 0.03	weight and density PCL + Ag vs. BCP/PCL + Ag *p* < 0.0001
BCP/PCL + Ag	NaCl	18.94 ± 0.06	9.99 ± 0.43	574.5 ± 39	0.20 ± 0.01	
**B**						
PCL	NaNO_3_	17.88 ± 0.06	10.10 ± 0.57	348.1 ± 25.85	0.14 ± 0.01	weight and density PCL vs. BCP/PCL *p* < 0.0001
BCP/PCL	NaNO_3_	17.86 ± 0.11	9.99 ± 0.86	574.2 ± 76.89	0.23 ± 0.02	
PCL + Ag	NaNO_3_	19.00 ± 0.13	10.88 ± 0.57	350.6 ± 46.03	0.11 ± 0.01	weight and density PCL + Ag vs. BCP/PCL + Ag *p* < 0.0001
BCP/PCL + Ag	NaNO_3_	18.69 ± 0.23	10.26 ± 0.64	546.8 ± 33.88	0.19 ± 0.03	

Abbreviations. PCL: poly(ε-caprolactone); NaCl: sodium chloride; NaNO_3_: sodium nitrate; BCP: biphasic calcium phosphate; Ag: silver.

## Data Availability

The source data underlying tables and figures are available from the authors upon request.
